# Rethinking radiology AI benchmarks

**DOI:** 10.1093/radadv/umag022

**Published:** 2026-04-11

**Authors:** Yi Lin, Yuzhe Yang, George Shih, Yifan Peng

**Affiliations:** Department of Population Health Sciences, Weill Cornell Medicine, New York, NY, 10022, United States; Department of Computational Medicine, University of California, Los Angeles, CA, 90095, United States; Department of Radiology, Weill Cornell Medicine, New York, NY, 10065, United States; Department of Population Health Sciences, Weill Cornell Medicine, New York, NY, 10022, United States; Department of Radiology, Weill Cornell Medicine, New York, NY, 10065, United States

**Keywords:** benchmarking, evaluation, AI

## Introduction

**Figure 1:**
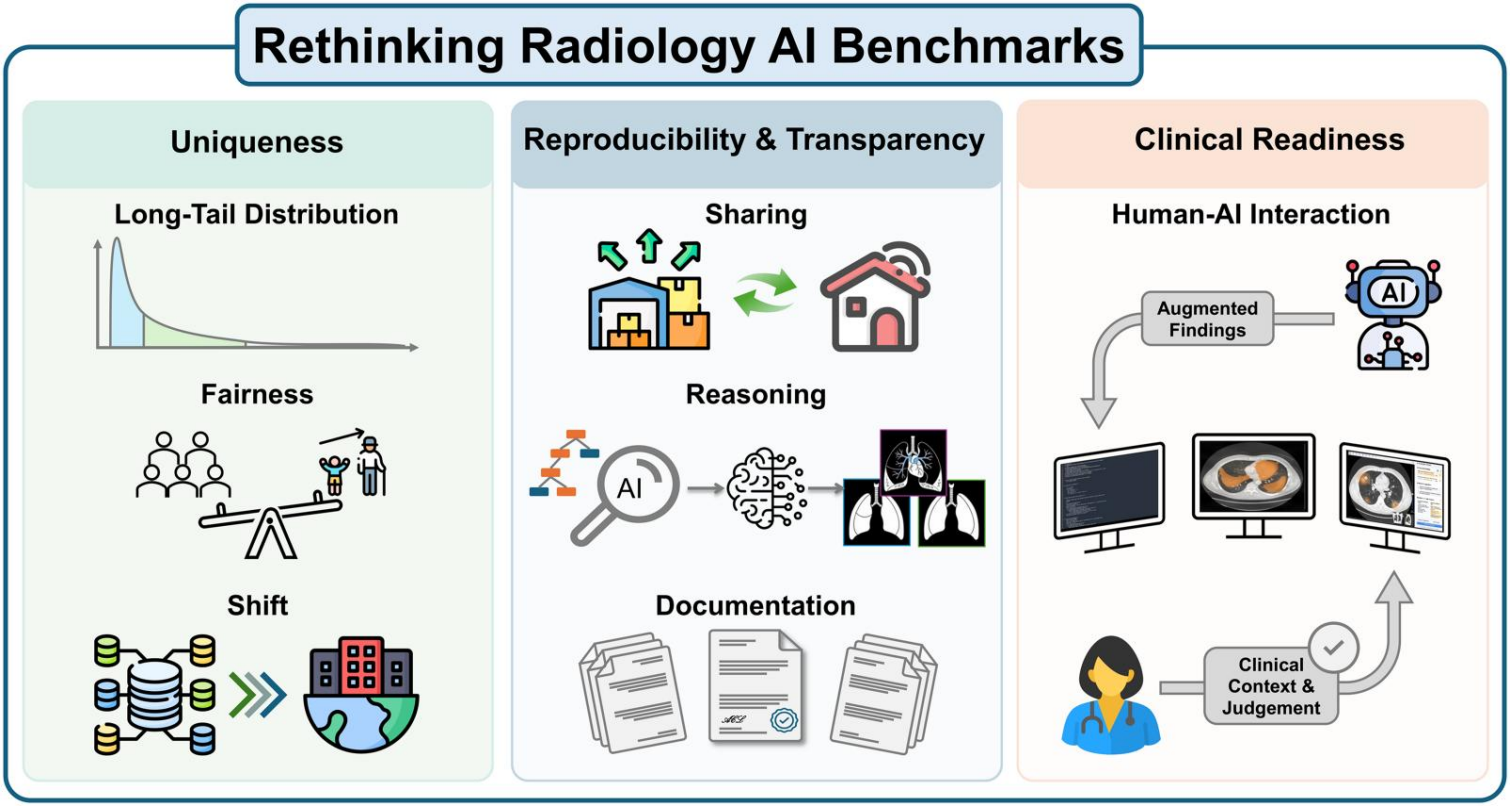
The 3 pillars of a rigorous and forward-looking radiology AI benchmark: uniqueness, transparency and reproducibility, and clinical readiness.

Public benchmarks have become a cornerstone for advancing artificial intelligence (AI) in radiology. In this context, a benchmark refers to a publicly available dataset paired with a standardized task, reference labels (ground truth), and an agreed-on evaluation protocol and metrics. Open imaging datasets create a common reference point across institutions and countries.^1^ They allow researchers to compare methods more fairly, reproduce findings more reliably, and build on prior work rather than starting from scratch. At the same time, the influence of benchmarks warrants careful consideration.^2^ Benchmarks do more than measure performance; they also shape research priorities and define what success looks like.^3^ If narrowly designed, they risk encouraging optimization for leaderboard rankings rather than meaningful clinical impact.^4^ For this reason, benchmarking efforts in radiology should be designed with both scientific rigor and clinical purpose (Table 1). In our view, a rigorous and forward-looking benchmarking study in radiology should demonstrate at least three essential elements: uniqueness, transparency and reproducibility, and clinical readiness (Figure 1).

**Table 1:** Three essential elements of a rigorous radiology AI benchmark and proposed improvements for clinical evaluation.

Essential element	Why it matters	Action items
Uniqueness	New evaluation value	Rare and high-severity cases; Tail-risk stress tests; Subgroup fairness analysis; Cross-site and cross-scanner robustness; Temporal robustness; Generative AI evaluation
Transparency and reproducibility	Trustworthy and comparable results	Clear cohort selection; Demographic and protocol reporting; Transparent preprocessing; Rigorous ground truth; Labeling workflow documentation; Adjudication process; Precise metric definitions; Shared evaluation code
Clinical readiness	Relevance to real practice	Calibration assessment; Severity-sensitive evaluation; Human-AI workflow studies; Reader simulations; Time-motion analysis; Multitask evaluation; Longitudinal assessment; Multimodal clinical context

Abbreviation: AI, artificial intelligence.

## Expanding what counts as uniqueness in radiology benchmarks

Many newly released radiology datasets are undoubtedly valuable. Yet a closer look shows that many are incremental extensions of what already exists. Simply increasing the number of participants and image studies does not automatically expand how we evaluate AI in radiology. To move forward meaningfully, future benchmarks must evolve along new evaluative dimensions.

### Stress-testing the tail

Radiologists often add the most value in rare, complex, or atypical cases. They are precisely where automated systems are most likely to fail. Most benchmark datasets reflect the natural distribution of disease, meaning common conditions dominate whereas rare diseases are underrepresented. As a result, a model can achieve high overall accuracy while failing consistently on uncommon conditions. From a clinical standpoint, this imbalance is concerning. Therefore, benchmarks need to reflect clinical priorities in rare-event challenge sets, subtle early-stage findings, and minimum performance requirements for high-severity conditions.

### Fairness

Fairness is another critical concern. Many radiology datasets provide limited information about participant characteristics such as age, sex, race, ethnicity, or socioeconomic background. Models are typically scored using aggregate metrics, which can hide uneven performance across subgroups.^5^ A future benchmark should require stratified performance reporting across clinically meaningful subgroups. Differences should be measured explicitly. For example, common fairness metrics such as demographic parity and equalized odds can be included alongside average accuracy to provide a fairer assessment.

### Distribution and temporal drift

A further limitation lies in insufficient evaluation under a distribution and temporal drift. First, everyday radiology is defined by its environment (eg, different scanners). A model that performs well in one may falter in another. Benchmarks should therefore conduct stress testing under intentional protocol variations, and measuring robustness to motion, noise, or incomplete studies should be routine. Second, clinical practice evolves continuously, diagnostic criteria are updated, and imaging protocols are changing. A model validated on historical data may silently degrade as these conditions change. Benchmarks should therefore encourage longitudinal reevaluation to assess whether model performance remains stable across time.

### Evaluating generative AI for radiology applications

The rapid emergence of generative AI introduces fundamentally different evaluation challenges. Unlike traditional image classifiers, generative AI produces free-style text and open-ended diagnostic reasoning. Conventional metrics such as AUC or Dice are ill-suited for such outputs. Natural language understanding metrics like BLEU and ROUGE can capture surface-level textual similarity but not clinical validity. Fluency does not ensure clinical validity. Benchmarks must therefore develop clinically grounded criteria for generative outputs, such as factual correctness and finding coverage. An equally pressing concern is data contamination: foundation models pretrained on large corpora may have already encountered public radiology datasets, leading to high scores that reflect memorization rather than genuine reasoning. Benchmark developers should document the release dates of datasets and implement contamination-detection protocols accordingly.

## Strengthening reproducibility and transparency

Reproducibility is fundamental to scientific progress. Yet in radiology AI research, benchmarks frequently lack detailed reporting of data preprocessing, labeling workflows, and evaluation procedures. Such omissions weaken the long-term scientific value of published results.

### Central role of ground truth

The validity of any benchmark is fundamentally constrained by the quality of its ground-truth labels. In radiology, labels are rarely self-evident. Diagnostic categories often depend on expert interpretation and may be confirmed by pathology, laboratory findings, and other outcomes. As a result, label curation is complex and context-dependent. When the labeling process is poorly defined, reported performance becomes less interpretable. Consequently, benchmark development must prioritize rigorous and transparent construction of ground truth. Without this foundation, subsequent performance comparisons lack scientific meaning.

### Transparency in dataset construction

Transparent reporting of dataset characteristics and preprocessing decisions is essential for reproducibility. Benchmarks should explicitly document key characteristics, such as inclusion and exclusion criteria, demographic composition, imaging protocols, and data sources. Equally important is clear documentation of preprocessing pipelines. Decisions such as normalization methods, handling missing data, augmentation strategies, and class-imbalance mitigation should be described as they can materially affect model performance.

### Documentation of labeling workflows

Beyond dataset description, detailed reporting of labeling workflows is critical. Key questions include: Were labels assigned by single or multiple experts? What were the experience levels of annotators? Was adjudication performed in cases of disagreement? Benchmarks should also report interreader variability, which provides an estimate of human-level agreement. Confidence intervals around labels or probabilistic annotations are more likely to accurately reflect clinical reality than binary assignments.

### Standardization and sharing of evaluation procedures

Finally, evaluation metrics must be defined precisely. For example, we should clarify whether results are macro- or micro-averaged when reporting multilabel classification results and specify the method to calculate overlap in segmentation tasks. To increase comparability across studies, the evaluation code should also be shared.

## Moving from benchmark scores to clinical readiness

As radiology AI systems move closer to clinical implementation, evaluation frameworks must evolve accordingly. Traditional benchmarks emphasize standalone performance metrics, often derived from retrospective datasets. However, clinical readiness requires more than high scores. Meaningful evaluation must extend beyond static benchmarks to include calibration, workflow integration, and clinical complexity.

### Evaluating calibration, not only discrimination

Most radiology AI benchmarks prioritize discrimination: how well a model separates positive from negative cases. Metrics such as AUC and Dice score capture ranking ability but ignore calibration. Calibration, which assesses whether a model’s predicted probabilities correspond to actual likelihoods, is critical for clinical decision-making. In triage systems, overconfident false positives can foster automation bias, encouraging clinicians to overtrust incorrect predictions. Conversely, underconfidence may reduce adoption or prompt unnecessary verification, negating efficiency gains. In clinical care, probabilistic predictions are interpreted as indicators of risk. Therefore, evaluation frameworks should assess whether predicted likelihoods align with observed outcomes. Moreover, not all errors carry equal consequences. Misclassification of a life-threatening condition should weigh more heavily than an incorrect benign label. Future benchmarks should incorporate severity-sensitive evaluation strategies that reflect the asymmetric costs of clinical errors.

### Evaluating the human-AI system

Current benchmarks typically assess models in isolation, detached from the environments in which they will operate. However, radiology is inherently workflow driven and team based. An algorithm that performs well independently may nonetheless introduce inefficiencies or cognitive burdens when integrated into practice. For example, a highly accurate system may slow reporting if its outputs require cumbersome verification. Poor interface design can further create distraction or alert fatigue. The clinically meaningful unit of analysis is therefore the combined human-AI system. The relevant question is not “How accurate is the model?” but rather, “Does the integrated human-AI team outperform either alone?” Evaluation strategies should include simulated reading-room studies, time-motion analyses, and comparative trials of AI-first versus radiologist-first workflows.

### Evaluating clinical complexity

Radiologic practice is inherently complex and cannot be reduced to a set of isolated classification tasks. In real clinical workflows, radiologists detect abnormalities, characterize findings, compare with prior studies, integrate laboratory data, and formulate management recommendations. These steps require continuous contextual reasoning rather than single-image interpretation. However, most current AI benchmarks focus on narrowly defined retrospective tasks using clean labels and static images. Such simplified setups fail to capture the complexity of real clinical environments and may overestimate the readiness of AI systems for clinical deployment. Additionally, relatively few benchmarks assess key aspects of clinical reasoning. To better reflect real clinical environments, future benchmarks should incorporate multitask evaluation frameworks, longitudinal case series, and multimodal clinical context integration. These approaches would more closely approximate the cognitive demands of the radiology reading room and provide a more meaningful assessment of AI systems intended for clinical use.

## Conclusion

The radiology community stands at a pivotal moment. Extraordinary technical gains on curated datasets have demonstrated what radiology AI can achieve, but leaderboard rankings and single-number metrics alone do not guarantee clinical value. The next generation of benchmarks should not be limited to larger or newer datasets; it should center on broader evaluation frameworks that reflect real-world medical complexity and patient-centered priorities. Bridging the gap from benchmark to bedside is not a question of algorithmic capability, but of redefining success through interdisciplinary collaboration and more comprehensive validation, ensuring that radiology AI advances the foundations of clinical care.
